# Supplementary material for: A Novel Approach to Assessing In-Hospital Mortality After On-Pump Aortic Valve Replacement
Source: Life (Basel). 2025 Oct 31;15(11):1696. doi: 10.3390/life15111696 (PMC12653218; doi:10.3390/life15111696)
Supplement: Supplementary file 1 [file life-15-01696-s001.zip › life-3934081-supplementary.pdf]

Supplementary Materials

Table S1. Type of on-pump SAVR

Type of surgery	N (%)
SAVR	214 (50.71%)
SAVR+CABG	65 (15.40%)
SAVR+AAS	63 (14.91%)
SAVR+ARE	39 (9.24%)
SAVR+MV	17 (4.02%)
SAVR+AAS+CABG	9 (2.13%)
SAVR+MV+CABG	2 (0.47%)
SAVR+ARE+AAS	2 (0.47%)
SAVR+ARE+MV	2 (0.47%)
SAVR+AAS+TV	2 (0.47%)
SAVR+MV+TV	2 (0.47%)
SAVR+ARE+CABG	2 (0.47%)
SAVR+CABG+MV+TV	2 (0.47%)
SAVR+ARE+MV+TV	1 (0.23%)

Abbreviation: AAS, ascending aorta surgery; ARE, aortic root enlargement; CABG, coronary artery bypass graft; MV, mitral valve; SAVR, surgical aortic valve replacement; TV, tricuspid valve

Table S2. Patient data reviewed in the 2 subgroups.

**Variable**	**Survivors (n=405)**	**Non-survivors (n=17)**	***p*[1]**
Male sex [2]	254 (62.71%)	9 (52.94%)	0.450
Age (years) [3]	66 [58-70]	65 [58-70.5]	0.670
Severe AS [2]	337 (83.20%)	15 (88.23%)	0.750
Preop AF [2]	39 (9.62%)	2 (11.76%)	0.676
COPD [2]	91(22.46%)	6 (35.29%)	0.241
DM [2]	113 (27.9%)	3 (17.64%)	0.421
HTN [2]	314 (77.53%)	11 (64.7%)	0.240
Clear_preop_creat (ml/min) [3]	86.72 [68.9-111.8]	83.82 [68.39-93.75]	0.426
BMI (kg/m2) [3]	28 [24.41-32.11]	30.38 [21.67-32.98]	0.440
Bicuspid AV [2]	121 (29.9%)	5 (29.41%)	1
LVEF (%) [3]	60 [55-60]	50 [50-60]	0.070
EuroSCORE [3]	6 [4-7]	7 [5.5-9]	0.001
EuroSCORE II [3]	1.51 [1.07-2.45]	3.4 [2.63-4.6]	0.001
Hb_preop (g/dl) [3]	13.5 [12.4-14.6]	13.2 [11.75-14.45]	0.458
Complex surgery [2]	194 (47.9%)	14 (82.35%)	0.006
Bioprosthetic AV 2	270 (66.67%)	12 (70.58%)	1
VIS [3]	4.3 [0-9]	27 [15.5-39]	0.001
Intraop_time (hours) 3	5 [4-5]	6 [6-8.5]	0.001
CPB_time (min) 3	95 [80-156.8]	174 [151.6-232]	0.001
ACC_time (min) [3]	72 [60-90]	128 [100.5-142]	0.001
RDW-SD_Preop (fl) [3]	42.8 [40.5-45.15]	42.6 [41.1-44.65]	0.953
PDW_Preop (fl) [3]	12.9 [11.65-14.35]	13.5 [12.3-15.15]	0.172
MPV_Preop (fl) [3]	10.8 [10.2-11.5]	11.1 [10.45-12]	0.131
L_Preop (*10^3/μL) [3]	7.41 [6.31-8.88]	8.79 [6.39-9.43]	0.164
N_Preop (*10^3/μL) [3]	4.6 [3.73-5.8]	5.57 [4.48-6.35]	0.062
M_Preop (*10^3/μL) [3]	0.58 [0.47-0.73]	0.59 [0.43-0.71]	0.812
P_Preop (*10^3/μL) [3]	226 [192.5-273]	229 [195.5-264.5]	0.806
Lf_Preop (*10^3/μL) [3]	1.93 [1.48-2.39]	1.99 [1.34-2.32]	0.957

SIRI_Preop [3]	1.39 [0.93-1.97]	1.55 [1.09-2.59]	0.327
AISI_Preop [3]	307.56 [210.21-475.15]	335.36 [230.72-543.78]	0.339
SII_Preop [3]	570.5 [385.41-778.75]	590 [404.32-998.97]	0.301
NLR_Preop [3]	2.34 [1.76-3.39]	3.1 [2.23-3.78]	0.178
MLR_Preop [3]	0.29 [0.23-0.36]	0.32 [0.18-0.44]	0.960
PLR_Preop [3]	120.58 [93.34-156.91]	125.83 [87.11-156.40]	0.849
RDW-SD_0 (fl) [3]	42.3 [40-44.75]	44.4 [41.95-48.5]	0.010
PDW_0 (fl) [3]	12.5 [11.15-14.05]	13 [12-14.7]	0.128
MPV_0 (fl) [3]	10.8 [10.2-11.5]	11.2 [10.4-11.75]	0.122
L_0 (*10^3/μL) [3]	12.74 [9.74-15.92]	15.27 [12.85-22.74]	0.011
N_0 (*10^3/μL) [3]	10.64 [7.8-13.59]	12.91 [9.78-18.07]	0.018
M_0 (*10^3/μL) [3]	0.77 [0.5-1.05]	0.91 [0.53-1.16]	0.388
P_0 (*10^3/μL) [3]	138 [112-172]	111 [86.5-127.5]	0.001
Lf_0 (*10^3/μL) [3]	1.16 [0.83-1.6]	1.64 [0.98-2.58]	0.038
SIRI_0 [3]	7.1 [3.68-11.25]	6.96 [4.74-13.25]	0.568
AISI_0 [3]	977.22 [515.47-1610.09]	817.44 [448.14-1629.64]	0.826
SII_0 [3]	1265.14 [872.33-1843.63]	844.8 [615.92-1576.47]	0.077
NLR_0 [3]	9.01 [6.71-12.77]	8.76 [6.79-11.26]	0.775
MLR_0 [3]	0.65 [0.41-0.96]	0.59 [0.35-0.96]	0.678
PLR_0 [3]	122.5 [85.83-166.33]	65.85 [36.35-118.96]	0.001
RDW-SD_1 (fl) [3]	43.7 [41.2-46.2]	44.5 [43.5-48.2]	0.037
PDW_1 (fl) [3]	13.4 [12.15-15.2]	15 [12.3-16.25]	0.212
MPV_1 (fl) [3]	11.3 [10.7-12]	11.9 [11.05-12.8]	0.045
L_1 (*10^3/μL) [3]	11.85 [9.44-14.24]	11.10 [9.09-14.94]	0.949
N_1 (*10^3/μL) [3]	9.67 [7.7-11.93]	9.58 [7.56-12.84]	0.976
M_1 (*10^3/μL) [3]	0.96 [0.74-1.29]	0.92 [0.6-1.19]	0.416
P_1 (*10^3/μL) [3]	155 [127-189.5]	121 [89.5-162.5]	0.005
Lf_1 (*10^3/μL) [3]	0.91 [0.68-1.19]	0.86 [0.62-1.03]	0.584
SIRI_1 [3]	10.44 [6.87-16.12]	12.64 [7.08-23.16]	0.473
AISI_1 [3]	1621.26 [988.51-2674.57]	1959.96 [753.17-2637.91]	0.561
SII_1 [3]	1720.89 [1131.19-2546.66]	1227.5 [894.91-2977.05]	0.511
NLR_1 [3]	10.97 [7.85-15.06]	13.45 [8.46-17.93]	0.360
MLR_1 [3]	1.06 [0.83-1.41]	1.15 [0.88-1.76]	0.448
PLR_1 [3]	173.68 [129.63-239.44]	147.77 [97.87-237.06]	0.249
SIRI_1-SIRI_0 [3]	3.24 [-0.28-7.27]	2.34 [-2.2-7.37]	0.539
SIRI_1-SIRI_Preop [3]	8.89 [5.28-14.21]	10.47 [4.82-20.49]	0.553
SIRI_0-SIRI_Preop [3]	-8.6 [-13.1 - -5.01]	-8.9 [-14.60 - -7.3]	0.437
AISI _1-AISI_0 [3]	541.94 [40.96-1318.6]	428.26 [-244.30 – 1557.86]	0.569
AISI _1-AISI_Preop [3]	-2041.99 [-3249.64 – 1285.65]	-2277.99 [-3319.53 – 1138.16]	0.725
AISI _0-AISI_Preop [3]	541.94 [40.96-1318.6]	428.26 [-244.3 – 1557.86]	0.569
SII_1-SII_0 [3]	1671.41 [1085.69-2502.46]	1184.9 [848.06-2931.2]	0.502
SII_1-SII_Preop [3]	1082.9 [626.26-1740.13]	708.81 [210.20-2006.99]	0.272
SII_0-SII_Preop [3]	-528.37 [-732.6- -340.49]	-544.8 [-949.22- -356.92]	0.313
NLR_1-NLR_0 [3]	1.33 [-1.46 – 5.41]	4.06 [-1.43 – 5.78]	0.513
NLR_1-NLR_Preop [3]	8.25 [5.53-12.39]	9.36 [5.16-14.39]	0.697
NLR_0-NLR_Preop [3]	6.65 [4.24-10.03]	6.15 [3.64-7.75]	0.440
PLR_1-PLR_0 [3]	47.96 [2.62-103.82]	62.88 [29.8-139.85]	0.273
PLR_1-PLR_Preop [3]	48.71 [11.92-97.8]	6.03 [-13.32- 95.32]	0.198
PLR_0-PLR_Preop [3]	3.49 [-31.35- 35.74]	-28.72 [-67.28- -8.78]	0.003
MLR_1-MLR_0 [3]	0.39 [0.07-0.74]	0.45 [0.1-0.73]	0.928
MLR_1MLR_Preop [3]	0.73 [0.53-1.06]	0.78 [0.6-1.33]	0.513
MLR_0 MLR_Preop [3]	0.33 [0.1-0.64]	0.25 [0-0.63]	0.593
AKI [2]	104 (25.67%)	17 (100%)	0.001
Hemostasis R [2]	33 (8.14%)	10 (58.82%)	0.001
RBCs (units) [3]	0 [0-2]	5 [3-6]	0.001
FFP (units) [3]	0 [0-2]	10 [4-12]	0.001

1 p value in Mann-Whitney test or exact Fisher test; 2 data are presented as n (%); 3 data are presented as median [IQR]. Note that: "*" is a multiplication sign; _Preop refers to the preoperative value of the variable; _0 refers to the variable value measured at intensive care admission; _1 refers to the variable value from day one after surgery. Abbreviations: ACC_time, aortic cross clamping time; AISI, aggregate index of systemic inflammation; AKI, acute kidney injury; AV, aortic valve; BMI, body mass index; CPB, cardiopulmonary bypass; Clear_preop_creat, preoperative creatinine clearance; COPD, chronic obstructive pulmonary disease; DM, diabetes mellitus; FFP, fresh frozen plasma; Hb_preop, preoperative hemoglobin concentration; Hemostasis R, reintervention for hemostasis; HTN, hypertension; Intraop_time, duration of the surgery; L, leukocytes count; Lf, lymphocytes count; LVEF, ejection fraction of left ventricle; M, monocytes count; MLR, monocytes to lymphocyte ratio; MPV, Mean Platelet Volume; N, neutrophils count; NLR, neutrophils to lymphocyte ratio; p probability value; P, platelet count; PDW, Platelet Distribution Width; PLR, platelet to lymphocyte ratio; Preop AF, preoperative atrial fibrillation; RBCs, red blood cell concentrates; RDW-SD, Red blood cell Distribution Width - standard deviation; SII, systemic inflammatory index; SIRI, systemic inflammatory response index; VIS, early postoperative vasoactive-inotropic score.

Table S3. The post hoc tests studying the inflammatory indexes in the three studied perioperative moments

Variable	Variable combinations	Survivors (p^1)	Non-survivors (p^1)
PLR	PLR_0-PLR_Preop	0.342	0.010
	PLR_1-PLR_0	0.001	0.005
	PLR_1-PLR_Preop	0.001	0.093
NLR	NLR_0-NLR_Preop	0.001	0.001
	NLR_1-NLR_0	0.001	0.102
	NLR_1 - NLR_Preop	0.001	0.001
MLR	MLR_0 - MLR_Preop	0.001	0.004
	MLR_1 - MLR_0	0.001	0.002
	MLR_1 - MLR_Preop	0.001	0.001
SII	SII_0 - SII_Preop	0.001	0.011
	SII_1 - SII_0	0.001	0.055
	SII_1 - SII_Preop	0.001	0.001
SIRI	SIRI_0 - SIRI_Preop	0.001	0.001
	SIRI_1 - SIRI_0	0.001	0.246
	SIRI_1 - SIRI_Preop	0.001	0.001
AISI	AISI_0 - AISI_Preop	0.001	0.006
	AISI_1 - AISI_0	0.001	0.093
	AISI_1 - AISI_Preop	0.001	0.001

[1] p value in Wilcoxon Signed Test. Note that: _Preop refers to the preoperative value of the variable; _0 refers to the variable value measured at intensive care admission; _1 refers to the variable value from day one after surgery. Abbreviation: AISI, aggregate index of systemic inflammation; MLR, monocytes to lymphocyte ratio; NLR, neutrophils to lymphocyte ratio; PLR, platelet to lymphocyte ratio; p, probability value; SII, systemic inflammatory index; SIRI, systemic inflammatory response index

Table S4. The post hoc tests studying the blood cell count in the three studied perioperative moments

Variable	Variable combinations	Survivors (p[1])	Non-survivors (p[1])
Leukocytes	L_0-L_Preop	0.001	0.010
	L_1-L_0	0.001	0.001
	L_1-L_Preop	0.001	0.001
Neutrophils	N_0-N_Preop	0.001	0.001
	N_1-N_0	0.001	0.004
	N_1 - N_Preop	0.001	0.001
Monocytes	M_0 - M_Preop	0.001	0.004
	M_1 - M_0	0.001	0.981
	M_1 - M_Preop	0.001	0.001
Lymphocytes	Lf_0 - Lf_Preop	0.001	0.245
	Lf_1 - Lf_0	0.001	0.001
	Lf_1 - Lf_Preop	0.001	0.001
Platelets	P_0 - P_Preop	0.001	0.001
	P_1 - P_0	0.001	0.061
	P_1 - P_Preop	0.001	0.001

[1] p value in Wilcoxon Signed Test. Note that: _Preop refers to the preoperative value of the variable; _0 refers to the variable value measured at intensive care admission; _1 refers to the variable value from day one after surgery. Abbreviation: L, leukocytes; Lf, lymphocytes; M, monocytes; N, neutrophils; P, platelets

\

Table S5. The binary logistic regression analysis targeting in-hospital death (n=422)

Variable	Univariable			Multivariable	
	Exp(B)	OR (CI 95%)	*p*	OR (CI 95%)	*p*
Age (years)			0.434		
Prothesis type			0.737		
Hemostasis R	16.1	16.1 (5.753-45.08)	0.001		0.712
Preop AF			0.771		
COPD			0.227		
DM			0.360		
HTN			0.225		
Severe AS			0.588		
Bicuspid AV			0.967		
Complex surgery	5.076	5.076 (1.437-17.931)	0.012		0.844
RBCs (units)	1.945	1.945 (1.530-2.473)	0.001		0.865
FFP (units)	1.486	1.486 (1.302-1.697)	0.001	1.335 (1.068-1.669)	0.011
Intraop_time (hours)	2.765	2.765 (1.892-4.040)	0.001		0.115
CPB_time (min)	1.037	1.037 (1.024-1.050)	0.001		
ACC_time (min)	1.038	1.038 (1.024-1.053)	0.001		
Clear_preop_creat (ml/min)			0.295		
BMI (kg/m2)			0.355		
VIS	1.069	1.069 (1.042-1.097)	0.001	1.058 (1.007-1.112)	0.024
LVEF (%)	0.939	1.064 (1.003-1.129)	0.041		
EuroSCORE	1.479	1.479 (1.78-1.856)	0.001		
EuroSCORE II	1.683	1.683 (1.334-2.124)	0.001		0.785
Hb_preop (g/dl)			0.296		
RDW-SD_Preop (fl)			0.566		
PDW_Preop (fl)			0.118		
MPV_Preop (fl)			0.116		
L_Preop (*10^3/μL)			0.211		
N_Preop (*10^3/μL)			0.134		
M_Preop (*10^3/μL)			0.688		
P_Preop (*10^3/μL)			0.808		
Lf_Preop (*10^3/μL)			0.898		
RDW-SD_0 (fl)	1.099	1.099 (1.014-1.193)	0.022		0.913
PDW_0 (fl)			0.153		
MPV_0 (fl)			0.133		
L_0 (*10^3/μL)	1.106	1.106 (1.031-1.186)	0.005		
N_0 (*10^3/μL)	1.104	1.104 (1.022-1.192)	0.012		
M_0 (*10^3/μL)			0.121		
P_0 (*10^3/μL)	0.978	1.022 (1.008-1.036)	0.002	1.033 (1.002-1.064)	0.034
Lf_0 (*10^3/μL)	2.314	2.314 (1.324-4.045)	0.003	3.532 (1.507-8.278)	0.004
RDW-SD_1 (fl)			0.232		
PDW_1 (fl)	1.176	1.176 (1.005-1.376)	0.043		
MPV_1 (fl)	1.712	1.712 (1.1-2.664)	0.017		
L_1 (*10^3/μL)			0.486		
N_1 (*10^3/μL)			0.405		
M_1 (*10^3/μL)			0.876		
P_1 (*10^3/μL)	0.982	1.018 (1.006-1.029)	0.004		0.113
Lf_1 (*10^3/μL)			0.648		
SIRI_Preop			0.699		
AISI_Preop			0.907		
SII_Preop			0.335		
NLR_Preop			0.180		
MLR_Preop			0.867		
PLR_Preop			0.643		
SIRI_0			0.226		
AISI_0			0.778		
SII_0			0.912		

NLR_0	0.105
MLR_0	0.547
PLR_0	0.380
SIRI_1	0.582
AISI_1	0.399
SII_1	0.806
NLR_1	0.532
MLR_1	0.556
PLR_1	0.530
SIRI_1-SIRI_0	0.482
SIRI_1-SIRI_Preop	0.611
SIRI_0-SIRI_Preop	0.232
AISI _1-AISI_0	0.597
AISI _1-AISI_Preop	0.460
AISI _0-AISI_Preop	0.597
NLR_1-NLR_0	0.562
NLR_1-NLR_Preop	0.637
NLR_0-NLR_Preop	0.144
PLR_1-PLR_0	0.982
PLR_1-PLR_Preop	0.317
PLR_0-PLR_Preop	0.191
MLR_1-MLR_0	0.957
MLR_1-MLR_Preop	0.499
MLR_0-MLR_Preop	0.494
SII_1-SII_0	0.803
SII_1-SII_Preop	0.566
SII_0-SII_Preop	0.342
AKI	0.993

Note that: "*" is a multiplication sign; _Preop refers to the preoperative value of the variable; _0 refers to the value of the variable measured at intensive care admission; _1 refers to the variable value from day one after surgery. Abbreviations: ACC_time, aortic cross clamping time; AISI, aggregate index of systemic inflammation ; AV, aortic valve; AKI, acute kidney injury; AS, aortic stenosis; BMI, body mass index; CI, confidence interval; CPB, cardiopulmonary bypass; Clear_preop_creat, preoperative creatinine clearance; COPD, chronic obstructive pulmonary disease; DM, diabetes mellitus; FFP, fresh frozen plasma; Hb_preop, preoperative hemoglobin concentration; Hemostasis R, reintervention for hemostasis; HTN, hypertension; Intraop_time, duration of the surgery; LVEF, left ventricle ejection fraction; L, leukocytes count; M, monocytes count; MLR, monocytes to lymphocyte ratio; Lf, lymphocytes count; M, monocytes count ; MPV, Mean Platelet Volume; NLR, neutrophils to lymphocyte ratio; N, neutrophils count; OR, odds ratio; *p* probability value; P, platelet count; PDW, Platelet Distribution Width; PLR, platelet to lymphocyte ratio; Preop AF, preoperative atrial fibrillation; RBCs, red blood cell concentrates; RDW-SD, Red blood cell Distribution Width - standard deviation; SII, systemic inflammatory index; SIRI, systemic inflammatory response index; VIS, early postoperative vasoactive-inotropic score

Table S6. ROC analysis in SAVR group (endpoint in-hospital mortality)

Variable	ROC			Cut off		
	AUC	*p*	CI 95%	Value	Ss (%)	Sp (%)
VIS	0.929	0.001	0.889-0.968	13.5	88.2	86.7
RBCs (units)	0.891	0.001	0.800-0.968	2.5	82.4	85.2
CPB_time (min)	0.871	0.001	0.762-0.980	160.5	76.5	91.6
FFP (units)	0.864	0.001	0.741-0.986	6.5	76.5	96.5
Intraop_time (hours)	0.861	0.001	0.786-0.935	5.5	82.4	75.3
ACC_time (min)	0.839	0.001	0.740-0.938	109.5	76.5	85.7
EuroSCORE II	0.838	0.001	0.752-0.925	2.19	94.1	70.1
P_0 (*10^3/μL)	0.739	0.001	0.630-0.847	131.5	56.8	88.2
PLR_0	0.739	0.001	0.602-0.876	66.00	87.9	52.9
EuroSCORE	0.725	0.002	0.614-0.836	6.5	64.7	67.3
PLR_Preop- PLR_0	0.716	0.001	0.596-0.835	15.56	76.5	63.5
P_1 (*10^3/μL)	0.702	0.003	0.567-0.837	133.5	69.4	70.6
RDW-SD_0	0.685	0.001	0.574-0.796	40.75	100	33.6
L_0 (*10^3/μL)	0.682	0.010	0.543-0.820	19.55	47.1	88.9

N_0 (*10^3/μL)	0.669	0.016	0.532-0.807	12.23	70.6	63.7
RDW-SD_1	0.649	0.002	0.554-0.744	42.55	100	38.5
MPV_1 (fl)	0.644	0.026	0.517-0.770	10.95	88.2	37.3
N_Preop (*10^3/μL)	0.633	0.027	0.515-0.752	4.44	82.4	46.4
Age (years)		0.674				
Clear_preop_creat (ml/min)		0.389				
BMI (kg/m2)		0.496				
LVEF (%)		0.125				
Hb_preop (g/dl)		0.459				
RDW-SD_Preop (fl)		0.950				
PDW_Preop (fl)		0.142				
MPV_Preop (fl)		0.106				
L_Preop (*10^3/μL)		0.163				
M_Preop (*10^3/μL)		0.812				
P_Preop (*10^3/μL)		0.805				
Lf_Preop (*10^3/μL)		0.958				
PDW_0 (fl)		0.076				
MPV_0 (fl)		0.093				
M_0 (*10^3/μL)		0.378				
Lf_0 (*10^3/μL)		0.053				
PDW_1 (fl)		0.236				
L_1 (*10^3/μL)		0.954				
N_1 (*10^3/μL)		0.978				
M_1 (*10^3/μL)		0.441				
Lf_1 (*10^3/μL)		0.584				
SIRI_Preop		0.327				
AISI_Preop		0.358				
SII_Preop		0.357				
NLR_Preop		0.161				
MLR_Preop		0.965				
PLR_Preop		0.863				
SIRI_0		0.567				
AISI_0		0.831				
SII_0		0.085				
NLR_0		0.762				
MLR_0		0.701				
SIRI_1		0.532				
AISI_1		0.577				
SII_1		0.587				
NLR_1		0.415				
MLR_1		0.493				
PLR_1		0.330				
SIRI_1-SIRI_0		0.557				
SIRI_1-SIRI_Preop		0.607				
SIRI_0-SIRI_Preop_		0.422				
AISI _1-AISI_0		0.573				
AISI _1-AISI_Preop		0.735				
AISI _0-AISI_Preop		0.576				
NLR_1-NLR_0		0.517				
NLR_1-NLR_Preop		0.717				
NLR_0-NLR_Preop_		0.419				
PLR_1-PLR_0		0.220				
PLR_1-PLR_Preop		0.238				
MLR_1-MLR_0		0.924				
MLR_1-MLR_Preop		0.538				
MLR_0-MLR_Preop_		0.624				
SII_1-SII_0		0.579				
SII_1-SII_Preop		0.352				
SII_0-SII_Preop_		0.371				

Note that: “*” is a multiplication sign; _Preop refers to the preoperative value of the variable; _0 refers to the variable value measured at intensive care admission; _1 refers to the variable value from day one after surgery. Abbreviations: ACC_time, aortic cross clamping time; AISI, aggregate index of systemic inflammation; AUC, area under the curve; BMI, body mass index; CI, confidence interval CPB, cardiopulmonary bypass; Clear_preop_creat, preoperative creatinine clearance; FFP, fresh frozen plasma; Hb_preop, preoperative hemoglobin concentration;; Intraop_time, duration of the surgery; L, leukocytes count; Lf, lymphocytes count; LVEF, ejection fraction of left ventricle; M, monocytes count; MLR, monocytes to lymphocyte ratio; MPV, Mean Platelet Volume; N, neutrophils count; NLR, neutrophils to lymphocyte ratio; p probability value; P, platelet count; PDW, Platelet Distribution Width; PLR, platelet to lymphocyte ratio; Preop AF, preoperative atrial fibrillation; RBCs, red blood cell concentrates; RDW-SD, Red blood cell Distribution Width - standard deviation; SII, systemic

inflammatory index; SIRI, systemic inflammatory response index; Sp, specificity; Ss, sensitivity; VIS, early postoperative vasoactive-inotropic score.

Table S7. ROC analysis in AS_SAVR group (n=320)

Variable	ROC			Cut off		
	AUC	*p*	CI 95%	Value	Ss (%)	Sp (%)
VIS	0.943	0.001	0.905-0.980	13.5	86.7	89.6
RBCs (units)	0.883	0.001	0.781-0.985	2.5	80	86.4
CPB_time (min)	0.870	0.001	0.748-0.991	141.5	80	88.7
Intraop_time (hours)	0.867	0.001	0.789-0.945	5.5	80	78.6
FFP (units)	0.849	0.001	0.713-0.986	6.5	73.3	97
EuroSCORE II	0.847	0.001	0.752-0.941	2.19	93.3	72.8
ACC_time (min)	0.837	0.001	0.726-0.947	109.5	73.3	88.4
PLR_0	0.814	0.001	0.701-0.927	66	89.9	60
PLR_Preop-PLR_0	0.775	0.001	0.685-0.865	15.56	86.7	65.3
EuroSCORE	0.753	0.001	0.632-0.874	6.5	73.3	68
P_0 (*10^3/μL)	0.737	0.001	0.614-0.861	131.5	56.1	86.7
L_0 (*10^3/μL)	0.729	0.001	0.592-0.866	19.53	53.3	88.7
Lf_0 (*10^3/μL)	0.725	0.001	0.590-0.859	1.63	60	77.4
N_0 (*10^3/μL)	0.710	0.002	0.574-0.846	15.72	53.3	85.5
RDW-SD_0 (fl)	0.691	0.001	0.576-0.807	40.75	100	33.5
P_1 (*10^3/μL)	0.676	0.023	0.525-0.828	133.5	68	66.7
N_Preop (*10^3/μL)	0.676	0.003	0.560-0.792	4.44	93.3	44.6
SII_0	0.660	0.026	0.519-0.800	821.03	79.2	53.3
L_Preop (*10^3/μL)	0.658	0.017	0.529-0.788	8.13	73.3	60.2
RDW-SD_1 (fl)	0.646	0.005	0.544-0.748	42.55	100	38.9
PLR_1-PLR_0	0.645	0.014	0.530-0.759	42.31	80	49.6
MPV_1 (fl)		0.136				
Age (years)		0.591				
Clear_preop_creat (ml/min)		0.508				
BMI (kg/m2)		0.389				
EF (%)		0.165				
Hb_preop (g/dl)		0.448				
RDW-SD_Preop (fl)		0.855				
PDW_Preop (fl)		0.158				
MPV_Preop (fl)		0.159				
M_Preop (*10^3/μL)		0.799				
P_Preop (*10^3/μL)		0.844				
Lf_Preop (*10^3/μL)		0.494				
PDW_0 (fl)		0.158				
MPV_0 (fl)		0.226				
M_0 (*10^3/μL)		0.275				
PDW_1 (fl)		0.373				
L_1 (*10^3/μL)		0.546				
N_1 (*10^3/μL)		0.591				
M_1 (*10^3/μL)		0.693				
Lf_1 (*10^3/μL)		0.867				
SIRI_Preop		0.339				
AISI_Preop		0.381				
SII_Preop		0.435				
NLR_Preop		0.301				
MLR_Preop		0.090				
PLR_Preop		0.082				
SIRI_0		0.832				
AISI_0		0.610				
NLR_0		0.067				
MLR_0		0.076				
SIRI_1		0.550				
AISI_1		0.725				
SII_1		0.568				
NLR_1		0.436				
MLR_1		0.667				
PLR_1		0.225				
SIRI_1-SIRI_0		0.979				
SIRI_1-SIRI_Preop		0.625				
SIRI_0-SIRI_Preop_		0.663				

AISI _1-AISI_0	0.958
AISI _1-AISI_Preop	0.899
AISI _0-AISI_Preop	0.958
NLR_1-NLR_0	0.137
NLR_1-NLR_Preop	0.727
NLR_0-NLR_Preop_	0.185
PLR_1-PLR_Preop	0.203
MLR_1-MLR_0	0.306
MLR_1-MLR_Preop	0.664
MLR_0-MLR_Preop_	0.241
SII_1-SII_0	0.561
SII_1-SII_Preop	0.345
SII_0-SII_Preop_	0.446

Note that: "*" is a multiplication sign; _Preop refers to the preoperative value of the variable; _0 refers to the variable value measured at intensive care admission; _1 refers to the variable value from day one after surgery. Abbreviations: ACC_time, aortic cross clamping time; AISI, aggregate index of systemic inflammation; AUC, area under the curve; BMI, body mass index; CI, confidence interval CPB, cardiopulmonary bypass; Clear_preop_creat, preoperative creatinine clearance; FFP, fresh frozen plasma; Hb_preop, preoperative hemoglobin concentration;; Intraop_time, duration of the surgery; L, leukocytes count; Lf, lymphocytes count; LVEF, ejection fraction of left ventricle; M, monocytes count; MLR, monocytes to lymphocyte ratio; MPV, Mean Platelet Volume; N, neutrophils count; NLR, neutrophils to lymphocyte ratio; p probability value; P, platelet count; PDW, Platelet Distribution Width; PLR, platelet to lymphocyte ratio; Preop AF, preoperative atrial fibrillation; RBCs, red blood cell concentrates; RDW-SD, Red blood cell Distribution Width - standard deviation; SII, systemic inflammatory index; SIRI, systemic inflammatory response index; Sp, specificity; Ss, sensitivity; VIS, early postoperative vasoactive-inotropic score
